# Evaluation of electromagnetic and nuclear scattering models in GATE/Geant4 for proton therapy

**DOI:** 10.1002/mp.13472

**Published:** 2019-04-15

**Authors:** Andreas F. Resch, Alessio Elia, Hermann Fuchs, Antonio Carlino, Hugo Palmans, Markus Stock, Dietmar Georg, Loïc Grevillot

**Affiliations:** ^1^ Division Medical Radiation Physics, Department of Radiotherapy, Christian Doppler Laboratory for Medical Radiation Research for Radiation Oncology Medical University of Vienna/AKH Wien Währinger Gürtel 18‐20 1090 Vienna Austria; ^2^ MedAustron Ion Therapy Centre/EBG MedAustron Marie‐Curie‐Straße 5 2700 Wiener Neustadt Austria; ^3^ Medical Radiation Science National Physical Laboratory Hampton Road TW11 0LW Teddington UK

**Keywords:** Monte Carlo simulations, proton beam therapy, dosimetry

## Abstract

**Purpose:**

The dose *core* of a proton pencil beam (PB) is enveloped by a low dose area reaching several centimeters off the central axis and containing a considerable amount of the dose. Adequate modeling of the different components of the PB profile is, therefore, required for accurate dose calculation. In this study, we experimentally validated one electromagnetic and two nuclear scattering models in GATE/Geant4 for dose calculation of proton beams in the therapeutic energy window (62–252 MeV) with and without range shifter (RaShi).

**Methods:**

The multiple Coulomb scattering (MCS) model was validated by lateral dose *core* profiles measured for five energies at up to four depths from beam plateau to Bragg peak region. Nuclear *halo* profiles of single PBs were evaluated for three (62.4, 148.2, and 252.7 MeV) and two (97.4 and 124.7 MeV) energies, without and with RaShi, respectively. The influence of the dose *core* and nuclear *halo* on field sizes varying from 2–20 cm was evaluated by means of output factors (OFs), namely frame factors (FFs) and field size factors (FSFs), to quantify the relative increase of dose when increasing the field size.

**Results:**

The relative increase in the dose *core* width in the simulations deviated negligibly from measurements for depths until 80% of the beam range, but was overestimated by up to 0.2 mm in *σ* toward the end of range for all energies. The dose *halo* region of the lateral dose profile agreed well with measurements in the open beam configuration, but was notably overestimated in the deepest measurement plane of the highest energy or when the beam passed through the RaShi. The root‐mean‐square deviations (RMSDs) between the simulated and the measured FSFs were less than 1% at all depths, but were higher in the second half of the beam range as compared to the first half or when traversing the RaShi. The deviations in one of the two tested hadron physics lists originated mostly in elastic scattering. The RMSDs could be reduced by approximately a factor of two by exchanging the default elastic scattering cross sections for protons.

**Conclusions:**

GATE/Geant4 agreed satisfyingly with most measured quantities. MCS was systematically overestimated toward the end of the beam range. Contributions from nuclear scattering were overestimated when the beam traversed the RaShi or at the depths close to the end of the beam range without RaShi. Both, field size effects and calculation uncertainties, increased when the beam traversed the RaShi. Measured field size effects were almost negligible for beams up to medium energy and were highest for the highest energy beam without RaShi, but vice versa when traversing the RaShi.

## 1 Introduction

In this work, we follow the terminology of Gottschalk et al.^1^ to describe a pencil beam (PB) in proton therapy with a brief summary provided below. The Gaussian shaped central high dose region, the *dose core*, is dominated by multiple Coulomb scattering (MCS) of the primary protons in the target material. This central high dose region is enveloped by a non‐Gaussian low‐dose region, the *dose halo*, constituting a considerable fraction of the laterally integrated dose. It is deposited by secondary charged particles resulting from nuclear scattering and single large‐angle scattered primary particles.^1^, ^2^, ^3^, ^4^, ^5^ The beam *halo* extends in lateral direction up to approximately one third of the beam range and reaches its maximum contribution around midrange (“midrange bump”). The *halo* is enveloped by the *aura*, which contains a negligible fraction of the dose of the PB and is transferred by indirectly ionizing particles. Dose contribution due to scattering in the delivery hardware (treatment head) is referred to as *spray*, which is facility dependent. In this study *core* and *halo* refer to the dose inside and outside of the central Gaussian‐shaped dose region, respectively.

Semianalytical dose calculation methods such as PB algorithms typically treat the MCS part analytically and apply correction factors to account for nuclear scattering and single large‐angle Coulomb scattering.^6^, ^7^ Up to 15% correction for a single 214 MeV PB beam in water and in clinical scenarios an error up to 5% in absolute dose calculation when neglecting the *halo* were reported.^2^ In Monte Carlo (MC) dose calculations, individual particles are tracked down to zero kinetic energy simulating the physical interactions according to modeled or tabulated cross sections.^8^, ^9^ The accuracy of MC simulations is limited by the accuracy of the physical models and numerical implementation. Using a MC dose calculation method instead of a PB algorithm was found to be beneficial in clinical routine when applying a range shifter (RaShi) in combination with large air gaps, for oblique beams or heterogeneous media.^10^ In that study,^10^ six out of seven planes in an anthropomorphic phantom passed a 90% Gamma index analysis criterion (3%/3 mm) for their MC simulations compared to film measurements, whereas only three out of seven passed using the PB algorithm. Speed optimized MC algorithms^11^ dedicated for routine clinical use are usually benchmarked against general purpose MC particle transport algorithms such as FLUKA^8^ and Geant4.^9^ The latter are traditionally used during commissioning phase to support basic beam data generation required for treatment planning systems (TPSs)^12^, ^13^ allowing to reduce time consuming measurements.^14^

Experimental validation of those general purpose MC algorithms focused on MCS^15^ or effects of the *halo* 16, 17, 18 within a limited energy range. MCS scattering angles in GATE/Geant4 were found to be underestimated on average compared to measurements in various materials.^15^ In polystyrene, the most tissue equivalent material reported, the scattering angle was underestimated by about 5% –10% for target thickness to range ratios less than 70%. The dose *halo* can be validated by means of depth dose profiles at constant radii^17^ or lateral dose profiles at constant depths.^16^ Due to the presence of a high dose gradient, misalignment and volume averaging effects limit the significance of the results.^14^, ^17^ However, these dose profile validations are useful and necessary to understand more complex situations such as output factors (*OFs*), which are normalized to a certain field size (typically around 10 cm^4^, ^5^, ^11^, ^16^, ^18^). *OFs* such as field size factors (FSFs) and frame factors (FFs) quantify the relative increase of the dose due to increasing field size and are expected to be less sensitive to lateral misalignment due to the symmetry of the field.

This study aims at validating electromagnetic and nuclear scattering models implemented in GATE/Geant4 in the clinical relevant energy range (62.4–252.7 MeV) and relies on a beam model taking into account all nozzle elements.^19^ The MCS model was validated by lateral dose *core* measurements at up to four depths for five energies (62.4–252.7 MeV) aiming to supplement literature with data in water.^15^ To study the *halo* at energies lower and higher than 177 MeV,^17^ lateral dose profiles were compared to measurements for three energies (62.4, 148.2 and 252.7 MeV). Finally, *OFs’* (FSF and FF) modeling accuracy has been validated as a combined test of electromagnetic and nuclear scattering models. In addition to the open beam configuration, the simulation accuracy with RaShi in the beam was investigated for two energies (97.4 and 124.7 MeV).

## 2 Materials and methods

### 2.A. Measurements

**Figure 1:**
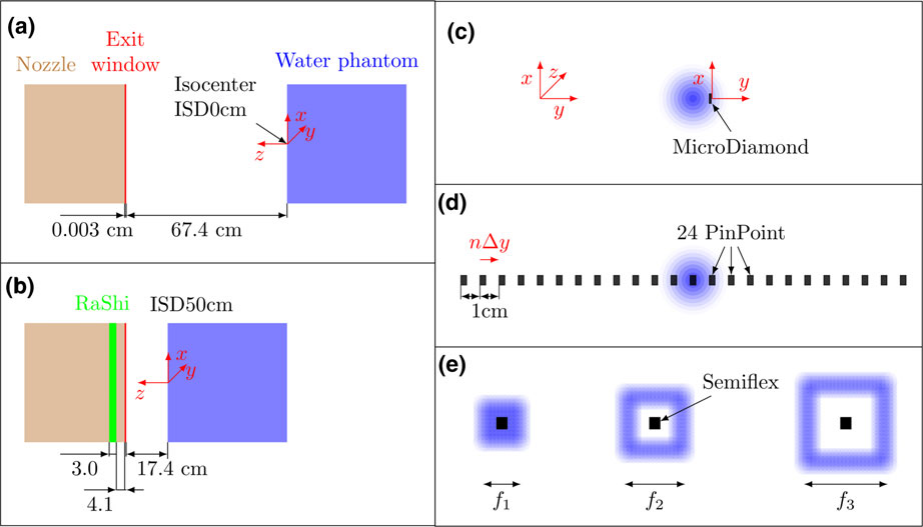
Overview of the measurement setup. The isocenter and ISD50cm setup is shown in (a) and (b), respectively. In (c–e), the *core*,* halo* and output factor (*OF*) measurements are sketched in beams’ eye view, respectively. (c) The *core* dose profile was measured by moving a MicroDiamond detector in vertical and horizontal direction at constant depths. (d) The *halo* dose profile of a single PB was measured by shifting 24 PinPoint ICs mounted on a linear array holder *n* times. (e) Frame fractors (FFs) were measured with a Semiflex IC at the center of hollow frames of varying size. [Color figure can be viewed at http://wileyonlinelibrary.com]

Figure 1, Table 1 and Table S1 (Supplementary Materials) give an overview of the measurement setup and of the used ionization chambers (ICs). GATE/Geant4 MC simulations were validated on three dose measurement sets, which can be grouped into the measurement of a single spot or a field of spots, at isocenter [Fig. 1(a)] and 50 cm isocenter to detector surface distance (ISD50cm) with RaShi [Fig. 1(b)]. All measurements were carried out in the horizontal beam line of treatment room 3 (IR3) at the MedAustron synchrotron facility using a remotely controlled water phantom MP3‐PL (PTW, Freiburg, Germany). Further details and descriptions of the equipment and calibration procedures are provided in the supplementary materials and literature.^20^, ^21^, ^22^ The detectors were positioned at the effective point of measurement $z_{\mathrm{ref}}$.^23^, ^24^

**Table 1:** Basic properties of the detectors used. The type number, the nominal active volume ($V_{A}$), and the nominal radius of the active volume ($R_{\mathrm{cav}}$) are given as listed by the manufacturer PTW (Freiburg, Germany)

Commercial name	Typenumber	Cross calibrated	Type	$V_{A}$ (cm${}^{3}$)	$R_{\mathrm{cav}}$ (mm)
Farmer	TM30013	Reference	Thimble IC	0.6	3.05
Semi‐flex	TM31010	No	Thimble IC	0.125	2.75
PinPoint	TM31015	Yes	Thimble IC	0.03	1.45
MicroDiamond	TM60019	No	Disc diamond diode	$4\cdot 10^{-6}$	1.10

*Lateral dose (core) profiles* were measured using the MicroDiamond (MD) detector due to its small sensitive area minimizing volume averaging effects. Beam profiles of five energies (62.4, 97.4, 148.2, 198.0, and 252.7 MeV) at isocenter without RaShi were measured in vertical and horizontal directions [see Fig. 1(c)].

*Lateral dose (halo) profiles* were determined using 24 PinPoint ICs in a linear array holder (PTW, Germany) with their cylinder axis perpendicularly oriented to the beam direction [see Fig. 1(d)]. The linear array was moved in the horizontal direction in steps of 2 (252.7 MeV) or 5 mm (62.4 and 148.2 MeV). Dose profiles of a single PB comprising $10^{10}$ initial protons for three energies (62.4, 148.2, and 252.7 MeV) at isocenter without RaShi and two energies (97.4 and 124.7 MeV) at ISD50 cm with RaShi were measured.

*The OFs* were measured with a Semiflex (SF) IC for frame sizes varying from 2 to 20 cm on the central axis at up to four depths. OFs were measured by decomposing a field of 20 cm × 20 cm into nine (hollow) square fields [see Fig. 1(e)], in the following referred to as frames. Defining a frame with minimum and maximum side lengths $a_{i}$ and $a_{o}$, a frame can be denoted as [$a_{i},a_{o}$] and the nine measured frame sizes were {[0,2.0], [2.4,3.2], [3.6,4.4], [4.8,5.6], [6.0,6.8], [7.2, 8.0,], [8.4, 10.0], [10.4, 15.6], [16.0,20.0]} cm. In this study, a particular frame is referred to by $a_{o}$. The smallest frame is not hollow, and will be referred to as in‐field measurement, whereas hollow rectangles will be referred to as out‐of‐field measurements. Note that the number of particles per PB ($10^{7}$) and the PB spacing (2 mm) were constant while the number of PBs per frame was not constant.

Frame sizes greater than 2 cm were measured in low dose range of the UNIDOS webline (T10021, PTW), whereas the smallest field size was measured in medium range.

### 2.B. Data analysis

*Lateral dose core* profiles were fitted with a Gaussian, $G(t|\mu ,\sigma^{2})$ using *lsqnonlin()* in MatlabR2016b (with the lateral coordinate *t*, the lateral Peak position *μ* and the standard deviation *σ*) in a two‐step process to avoid biasing by the non‐Gaussian dose contributions. Firstly, an initial guess on $\sigma_{\mathrm{init}}$ was derived from a fit using a 5% dose threshold. The final $\sigma_{C}$ was determined by another fit to all *t* within $2.5\sigma_{\mathrm{init}}$ as the lateral shape is Gaussian up to approximately 2.5*σ* for a high‐energy proton beam.^25^

The *core* width in water at depth *z*, $\sigma_{C}\left(z\right)$, can be expressed as the root of the squared sum of an initial width $\sigma_{C}\left(z_{0}\right)$, a MCS part $\sigma_{\mathrm{MCS}}\left(z\right)$ and a drift term, $\sigma_{d}^{2}\left(z,\theta_{0}\right)$, due to the initial divergence $\theta_{0}$ for $z\geq z_{0}$:

(1) $$\sigma_{C}^{2}\left(z\right)=\sigma_{C}^{2}\left(z_{0}\right)+\sigma_{\mathrm{MCS}}^{2}\left(z\right)+\sigma_{d}^{2}\left(z,\theta_{0}\right).$$

The transformation

(2) $$\sigma_{\mathrm{sc}}^{2}\left(z\right):=\sigma_{\mathrm{MCS}}^{2}\left(z\right)+\sigma_{d}^{2}\left(z,\theta_{0}\right)=\sigma_{C}^{2}\left(z\right)-\sigma_{C}^{2}\left(z_{0}\right)$$

makes $\sigma_{\mathrm{sc}}^{2}\left(z\right)$ independent of the initial beam width and only retains a dependence on the initial divergence at $z_{0}$, which was set to the first measurement depth at 14/20 mm depth in water. The beam divergence at $z_{0}$ depends on the initial beam divergence at the entrance of the water phantom *z* = 0, which are both experimentally unknown.

*Lateral dose halo profiles:* The full width at a fraction *x* of the maximum (FWxM) were derived from the profiles, where *x* was chosen to be 50%, 1%, 0.1%, and 0.05%. In this notation, the FWHM is represented by the FW50%M. FW1%M was derived by linear interpolation of the data, whereas all lower levels were derived from a linear exponential fit (*a* exp (−*bt*)) with two free parameters (*a*,* b*) to all data points within ±50% of the desired level *x*. Horizontal misalignment in the measurement was corrected for by applying the offset *μ* obtained via a $G(t|\mu ,\sigma^{2})$ fit. The horizontal offset *μ* was found to be up to 0.6 mm.

The 1*σ* confidence interval (CI) on dose measurement reproducibility was calculated from the square root of the sum of the squared contributions, namely, the standard deviation of the three repetitions and an assumed day to day absolute dose delivery reproducibility of 0.6%.

*The OFs* were evaluated by means of FFs^2^ and FSFs.^16^ The FF${}_{i}$ is the dose at the center of the field by frame *i* relative to the dose of a 10 cm × 10 cm field $D_{N}$, or formally:

(3) $$FF_{i}=\frac{D_{i}}{D_{N}}$$

with the dose $D_{i}$ and the normalization $D_{N}=\sum_{i=1}^{7}D_{i}$. The frame sizes of *i* = 7 and *i* = 9 are 10 and 20 cm, respectively. The FSF is the cumulative FF

(4) $$FSF_{i}=\frac{\sum_{k=1}^{i}D_{k}}{D_{N}}.$$

An uncertainty $\Delta D_{i}$ in the dose measurement propagates into an uncertainty

(5) $$\Delta FSF_{i}=\sqrt{\frac{\sum_{k=1}^{i}\Delta D_{k}^{2}}{D_{N}^{2}}+{\left(\frac{\sum_{k=1}^{i}D_{k}}{D_{N}^{2}}\Delta D_{N}\right)}^{2}}$$

with the uncertainty of the dose of the reference field $\Delta D_{N}=\sqrt{\sum_{i=1}^{7}\Delta D_{i}^{2}}$.

The root mean square deviation (RMSD) of all FSFs for fields larger than 4 cm (corresponds to *i* ≥ 3) was calculated at each depth in order to estimate the quality of agreement at a single depth:

(6) $$RMSD=\sqrt{\frac{\sum_{i=3}^{9}{\left(FSF_{i}^{\mathrm{MC}}-FSF_{i}^{\mathrm{meas}}\right)}^{2}}{7}}$$

The threshold of 4 cm was chosen such that FSFs are mostly dominated by the *halo* (in Fig. 6 we will see that the *core* contributes up to this field size for large spot sizes) and to be comparable to literature mostly reporting FSFs larger than 4 cm.^5^, ^18^

*To separate the core and halo contributions to FSFs*, FSFs only considering the *core* were analytically calculated using a Gaussian function. To simplify the interpretation of the results, a one‐dimensional Gaussian representing a radial distribution was used applying the horizontal $\sigma_{C}$ obtained from measurements and MC simulations. The sensitive area of the detector was subdivided into 0.1 mm × 0.1 mm bins and the energy deposition of all bins was summed up. The energy deposition in each bin was calculated from $G({\vec{r}}_{b}|{\vec{\mu }}_{\mathrm{PB}},\sigma_{C}^{2})$ with ${\vec{r}}_{b}$, the center of the bin, and ${\vec{\mu }}_{\mathrm{PB}}$, the PB position.

### 2.C. MC simulations

The MC simulations were carried out using GATE version 8.0^26^, ^27^ in combination with Geant4 10.03.p01 and a dedicated beam model including full simulation of the Nozzle elements. The material composition and the geometry of the individual elements were adopted from the blueprints and the latter was additionally verified by measurements wherever possible.^19^ The beam model was developed and validated using the reference hadron physics list^28^, ^29^ and the electromagnetic option 4, *EMZ*. Details on the physics models employed can be found in the Geant4 Physics Reference Manual.^30^ While the mean excitation potential is often considered a free parameter in order to match the measured ranges, it was fixed in our beam model a priori to 78 eV based on the recommendations in ICRU report 90.^31^ The range deviations were then minimized by lowering the initial energy resulting in a range agreement for all energies better than 0.2 and 0.35 mm without and with RaShi, respectively. The FWHM of the beam in air agreed with measurements within clinical requirement of 1 mm/10% at seven positions ranging from 58 cm upstream (ISD58cm) to 20 cm downstream the isocenter (ISD‐20cm). The production cut for secondary *e*−, *e*+ and *γ* was set to 10 mm outside the Nozzle and 1 m inside the Nozzle. The maximum step size was limited to 1 cm in the Nozzle and 100 μm in the water phantom and the RaShi. All scoring geometries in the simulations were positioned at depth $z_{\mathrm{ref}}$. All simulations were carried out in water neglecting the PMMA entrance window of the water tank as a negligible effect is expected on medium energy range nuclear interactions,^1^, ^32^ which was confirmed by a MC simulation for the medium energy beam.

In the *core* dose profile simulations, dose was scored in vertical and horizontal linear profiles with a 0.5 mm resolution, whereas cylindrical symmetry was exploited to increase statistics in the *halo* dose profile simulations using a virtual cylindrical scoring grid. Energy deposition was scored and normalized by the mass of the hollow cylinder ($(r_{o}^{2}-r_{i}^{2})\pi \Delta z\rho$, with the outer $r_{o}$ and inner radius $r_{i}$ and density *ρ*). To account for the volume averaging of the PinPoint ICs, the axial (Δ*z*) and radial (Δ*r*) scoring resolution was 2.5 mm (the side length of a square yielding a similar area as the circle with the nominal radius $R_{\mathrm{cav}}$ of the IC used in the measurements).

In the *OF* simulations, energy deposition was scored in a virtual cylinder with radius $R_{\mathrm{cav}}$ of the Semiflex (SF) IC centered at $z_{\mathrm{ref}}$ using the dimensions as in Table 1. Spot positions and frame sizes were identical to those in the measurements.

To separate the contributions of the beam model from the MCS model uncertainties when evaluating *core* dose deviations, three sets of MC simulations were carried out with non‐default settings. Equation (2) shows, that the beam width does not only depend on the MCS in water but also on the beam divergence before entering the water. In order to estimate the influence of the initial divergence component, the *core* profile was simulated with a generic beam starting at the surface of the water having approximately zero beam divergence and emittance at 252.7 MeV (first set of non‐default MC settings).

The second set of MC simulations with non‐default settings was carried out to rule out that the observed deviations in the *halo* profile with RaShi originated from impurities of the RaShi material. The RaShi consists of a 3 cm‐thick PMMA plate (Plexiglas^®^). For MC simulations, the material composition was adopted from PSTAR 33. By default, the mean excitation potential was 68 eV, calculated using Bragg’s rule.^30^ In order to evaluate the influence of the material composition, we simulated the dose *halo* of the 124.7 MeV beam using *I* = 74 MeV as tabulated 33 and a second simulation adding three mass percent of iron to PMMA (which supposedly only consist of hydrogen, carbon and oxygen) (I = 71 eV). For both simulations, the density was adapted within the density uncertainty to yield the correct WET of the RaShi. The differences in the *halo* were negligible.

In order to explain the physics origin of the observed deviations with RaShi, the default hadron physics list was replaced with *QGSP*_*BIC* (third set of non‐default MC settings). For readability only results, which differ from the default, are listed and described always at the end of each section. Additional data can be found in the supplementary material. The CHIPS and Barashenkov‐Glauber–Gribov (BGG) elastic scattering cross sections (CSs) are applied by default in *QGSP*_*BIC* and *QBBC*, respectively. Therefore, those CSs were extracted from Geant4 using *G4HadronicProcessStore* as provided in the Geant4 example “Hadr00”.

## 3 Results

### 3.A. Lateral dose *core* profiles

**Figure 2:**
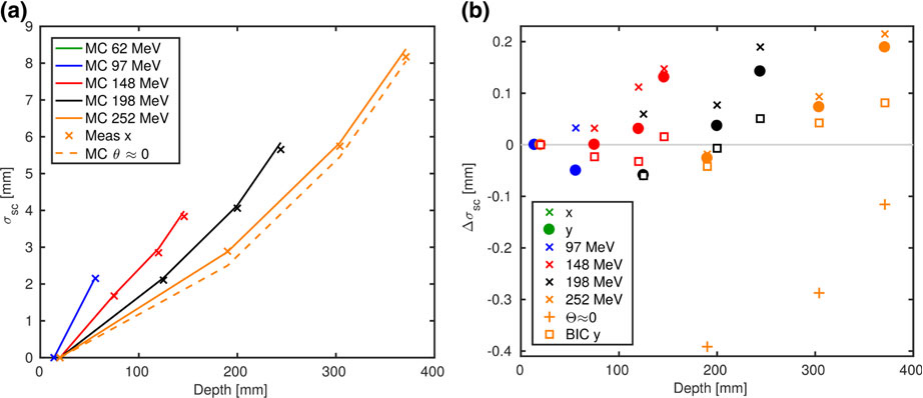
Increase of the pencil beam width in water relative to the width at reference depth ($\sigma_{\mathrm{sc}}$). Monte Carlo (MC) simulations and measurements are represented with lines and symbols, respectively. The vertical direction is plotted in (a) and the difference between MC and experiment of both directions in (b). The differences in horizontal direction using *BIC* are represented with squares. [Color figure can be viewed at http://wileyonlinelibrary.com]

The *core* width $\sigma_{C}$ agreed within −7% and +2% or −0.5 and +0.2 mm at all depths and for all energies. Table 2 lists measured $\sigma_{C}\left(z\right)$ and the deviations in the MC simulations. At the shallowest depth $z_{0}$, $\sigma_{C}\left(z_{0}\right)$ was underestimated in the MC simulations by −7% to −3%, whereas it was less underestimated or even overestimated at the end of range. To separate MCS uncertainties from beam modeling uncertainties, $\sigma_{\mathrm{sc}}$ is plotted in Fig. 2. Although the measured beam *core* profile ($\sigma_{C}$) was up to 0.3 mm asymmetric in horizontal and vertical direction at $z_{0}$, $\sigma_{\mathrm{sc}}$ was independent of the initial beam width, which allows us to only plot the vertical direction in Fig. 2(a). MC simulated $\sigma_{\mathrm{sc}}$ agreed with measurements until midrange, but were more and more overestimated toward the end of the range. At 97% of the beam range $\sigma_{\mathrm{sc}}$ was up to 0.2 mm overestimated.

**Table 2:** Core widths $\sigma_{C}\left(z\right)$ at several depths *z*. Both, measured $\sigma_{C}$ in horizontal direction, $\sigma_{y}^{\mathrm{meas}}$, and deviations in the Monte Carlo (MC) simulations $\Delta \sigma_{y}^{\mathrm{MC}}$ and $\Delta \sigma_{y}^{\mathrm{BIC}}$ using the default physics list and *BIC*, respectively, are given in millimeters

	62.4 MeV		148.2 MeV			252.7 MeV		
*z* a	$\sigma_{y}^{\mathrm{meas}}$	$\Delta \sigma_{y}^{\mathrm{MC}}$	$\sigma_{y}^{\mathrm{meas}}$	$\Delta \sigma_{y}^{\mathrm{MC}}$	$\Delta \sigma_{y}^{\mathrm{BIC}}$	$\sigma_{y}^{\mathrm{meas}}$	$\Delta \sigma_{y}^{\mathrm{MC}}$	$\Delta \sigma_{y}^{\mathrm{BIC}}$
$z_{0}$	9.09	−0.47	4.53	−0.30	−0.30	3.17	−0.23	−0.23
50%			4.84	−0.28	−0.29	4.31	−0.18	−0.19
80%			5.40	−0.23	−0.27	6.58	−0.04	−0.07
97%			5.96	−0.14	−0.19	8.80	0.10	−0.01

^a^Relative to the beam range in water (R80) or at 14/20 mm depth.

The beam model accuracy enters $\sigma_{\mathrm{sc}}$ by the beam divergence and emittance before entering the water phantom [see Eq. (2)] as the simulation with the non‐diverging beam confirms. The $\sigma_{\mathrm{sc}}$ using the non‐diverging beam resulted in notably smaller $\sigma_{C}$ and a shift of the deviations by −0.3 to −0.4 mm. While with default MC settings, highest deviations occurred at the Bragg peak, the highest deviations in the non‐divergent beam were found at midrange and decreasing thereafter.

Simulations using *QGSP*_*BIC* resulted in slightly lower $\sigma_{\mathrm{sc}}$ and deviations to measurements. Highest deviation at the end of the range was 0.1 mm.

### 3.B. Lateral dose *halo* profiles

**Figure 3:**
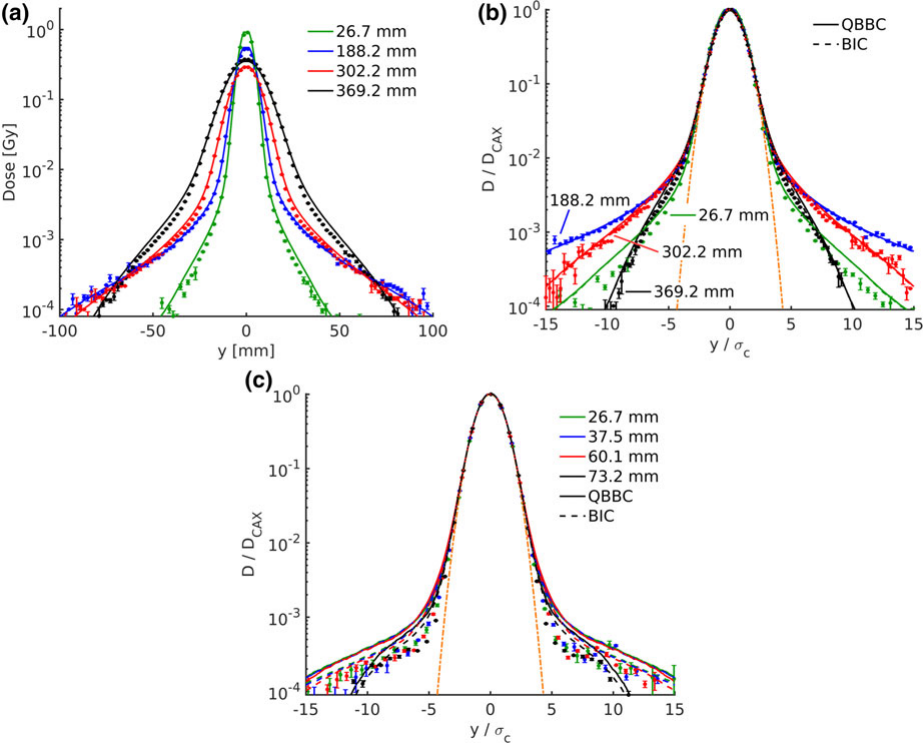
Simulated (lines) and measured (markers) lateral dose profiles of a central proton pencil beam at several depths in water. The 252.7 MeV beam profile without RaShi is shown in (a, b) and the 124.7 MeV beam profile with RaShi is shown in (c). Dose and lateral distance from the CAX are displayed in absolute values in (a), whereas dose is normalized to the CAX dose and lateral distance to $\sigma_{C}$ in (b,c). In (b, c), additionally, Monte Carlo (MC) simulations using the *BIC* are plotted as dashed lines. [Color figure can be viewed at http://wileyonlinelibrary.com]

Simulated lateral dose profiles agreed well with measurements in the *halo* region for all energies without RaShi. MC simulations modestly overestimated the dose in the *halo* region in the distal depth close to the Bragg peak. This effect was highest for the highest energy and is shown in Figs. 3(a) and 3(b). However, the *halo* was considerably overestimated in the MC simulations when the beam traversed the RaShi for both tested energies [see Fig. 3(c)].

The double normalized (to maximum dose and $\sigma_{C}$) representation in Figs. 3(b) and 3(c) facilitates to distinguish the Gaussian *core* and the *halo* and accounts for $\sigma_{C}$ deviations. The dose profile does not deviate from a Gaussian until approximately 2.5$\sigma_{C}$ for all energies and all depths as reported for a high‐energy proton beam.^25^ Furthermore, the dose *halo* is increasing with energy and as expected was relative to the central axis (CAX) dose highest around midrange. In the 252.7 MeV beam the dose can exceed 0.1% of the CAX dose up to distances 10 times $\sigma_{C}$, whereas only up to 7 $\sigma_{C}$ in the 148.2 MeV beam. The results for the 148.2 MeV beam can be found in the supplementary materials Fig. S1. The dose in the *halo* region was highest for the highest energy without RaShi, but inversely with RaShi, where the *halo* was higher for the lower energy.

**Figure 4:**
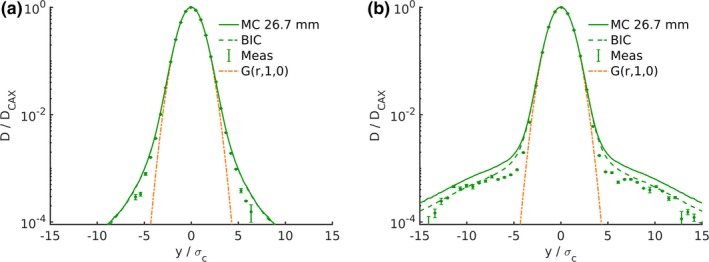
Simulated (lines) and measured (markers) lateral dose beam profiles of two pencil beams of similar residual range in water without (a) and with (b) RaShi. Dose was normalized to the maximum and the distance to the central axis is normalized to $\sigma_{C}$. Monte Carlo simulations using the *QGSP*_*BIC* are represented by dashed lines. [Color figure can be viewed at http://wileyonlinelibrary.com]

Figure 4 shows the lateral dose profile of two PBs with approximately the same range in water, one of the PBs passing through the RaShi. The increase of the dose *halo* for the beam traversing the RaShi is evident. The double normalized representation was chosen to account for the smaller beam size of the beam measured at ISD50cm (97.4 MeV with RaShi) compared to the beam measured at isocenter (62.4 MeV).

**Figure 5:**
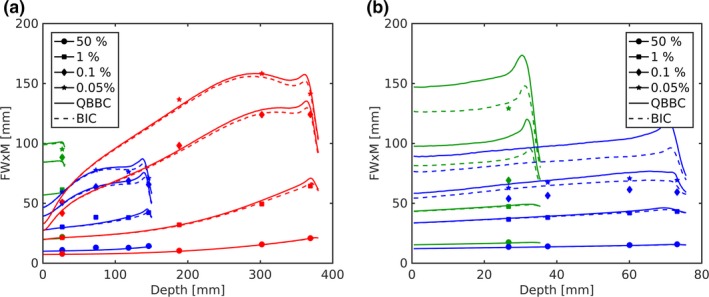
The measured and Monte Carlo simulated FWxM as a function of depth in water are plotted with markers and lines, respectively. In (a) the 62.4, 148.2, and 252.7 MeV pencil beams in the open beam configuration at isocenter are displayed in green, blue and red, respectively. In (b) the 97.4 and the 124.7 MeV at ISD50cm with RaShi are displayed in green and blue, respectively. [Color figure can be viewed at http://wileyonlinelibrary.com]

The FWxM as a function of depth is shown in Fig. 5 without (a) and with (b) RaShi. In the open beam configuration, FW at 50% and 1% of the maximum are monotonically increasing with depth until the peak, whereas FW0.1%M and FW0.05% start to reveal a maximum before the end of range for the higher energy. However, with RaShi the FW0.1%M and FW0.05% not only appear approximately 50% higher but also increased only by few percent over depth similar to the low‐energy beam without RaShi. As the FWxM (*x* < 1%) were almost constant over depth with RaShi, one can expect rather constant *OFs* over depth. Without RaShi MC simulations agreed within −4 to +6 mm (or −7 to +10%) with measured FWxM (*x* < 50%), whereas FW0.1%M and FW0.05% were up to 55% overestimated with RaShi.

The lateral dose profile using *QGSP*_*BIC* differed to the ones using *QBBC* in two ways: toward the end of the range of the two highest energies the intermediate dose region (approximately 3–7 $\sigma_{C}$) and the low dose area when the beam passes the RaShi were less overestimated [see Figs. 3(b), 3(c) and 4]. The FWxM distributions were vastly similar in the open beam configuration [see Fig. 5], but agreed better with measurements at depths starting from approximately 80% of the range. In the situation with RaShi, FWxM were considerably less overestimated using *QGSP*_*BIC* (up to 30%) compared to *QBBC* (up to 55%).

### 3.C. *OFs*

**Figure 6:**
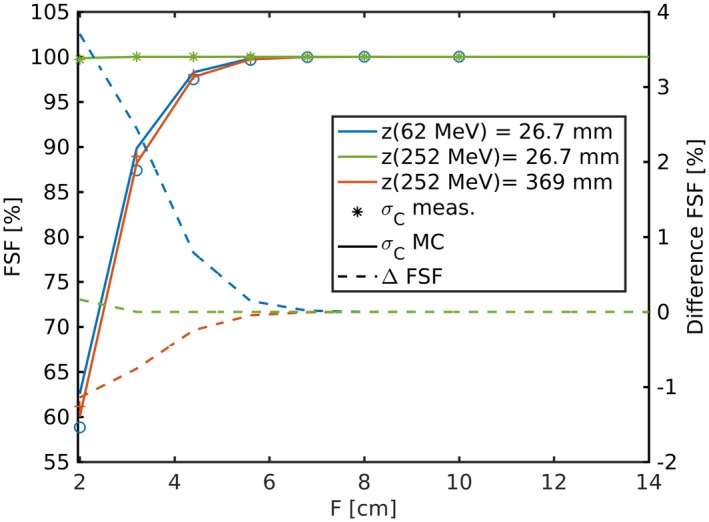
Analytically calculated field size factors (FSF) using $\sigma_{C,y}$ from the measurements (points) and Monte Carlo (MC) simulations (solid lines) of the 62.4 and 252.7 MeV proton beam. The corresponding differences are illustrated as dashed lines. [Color figure can be viewed at http://wileyonlinelibrary.com]

The FSFs for a hypothetical PB which would only comprise MCS described by a Gaussian distribution are shown in Fig. 6 using $\sigma_{C}$ obtained from MC simulations and measurement as listed in Table 2. FSFs would range from approximately 60 to 100% for the two biggest spot sizes, $\sigma_{C}$, while there would almost be no field size effect for the smallest $\sigma_{C}$ as the shortest distance between the surface of the IC and the closest PB of frame [2.4,3.2] is more than $3\sigma_{C}$. Even for the biggest spot sizes, which are of similar size (≈2 cm FWHM), FSFs would be negligible for fields bigger than 4.4 cm.

In the previous section, $\sigma_{C}$ was found to be underestimated in the MC simulations at the proximal plane of 62.4 and 252.7 MeV by −5% and −7%, respectively. This underestimation of the dose *core* results in an overestimation of FSFs (see Fig. 6) up to 3.7%. Although the relative spot size deviation at the proximal plane of the 252.7 MeV beam was higher, the deviation on FSFs were negligible due to the three times smaller spot size. At the end of the beam range $\sigma_{C}$ was found to be overestimated in the MC simulations by approximately 1%, which resulted in an up to −1.2% underestimation of FSFs. An underestimation of $\sigma_{C}$ means that the Gaussian *core* is too sharp and less dose of a distal frame reaches the detector and at the same time less dose of the in‐field (*F* = 2 cm) scatters out of the field. Vice versa for an overestimation of $\sigma_{C}$, too many protons of the inner field scatter out of the inner field and more protons from distal layers reach the detector.

**Figure 7:**
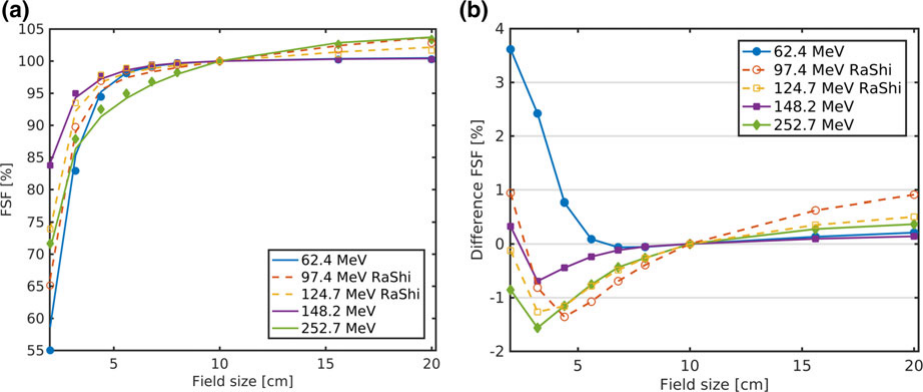
(a) FSFs at 80% and 89% of the beam range of the high (124.7, 148.2, and 252.7 MeV) and the low energies (62.4 and 97.4 MeV), respectively. MC simulated FSF are represented with lines and measurements with symbols. (b) The corresponding differences of MC simulated and measured FSFs. [Color figure can be viewed at http://wileyonlinelibrary.com]

**Figure 8:**
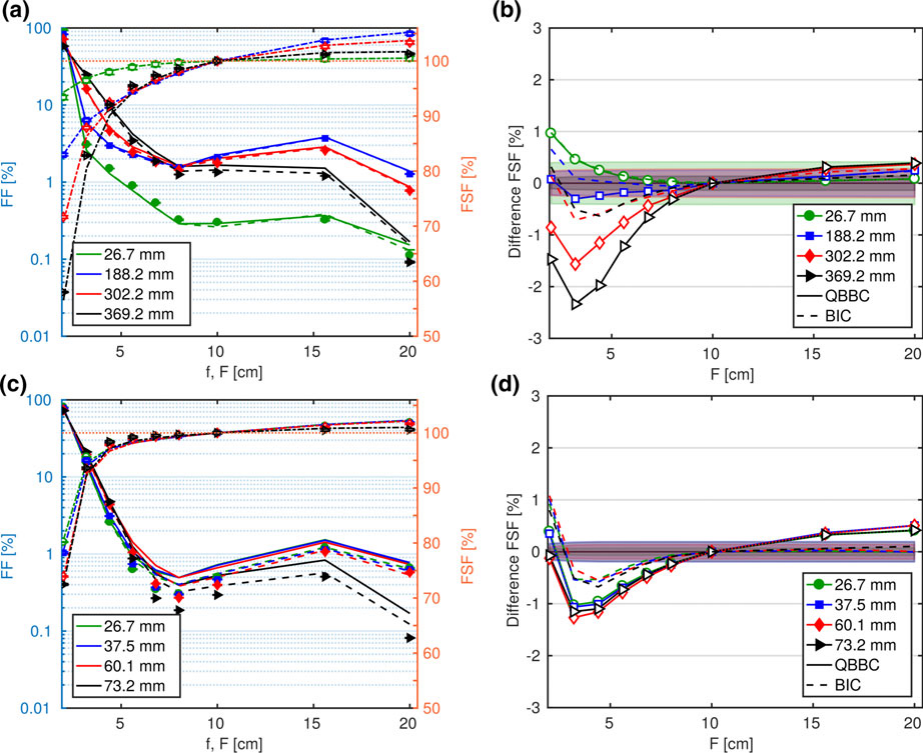
Frame factors (FFs) and field size factors (FSFs) as a function of field sizes for the 252.7 and 124.7 MeV in (a) and (c), respectively. Monte Carlo simulations are represented with lines and measurements with symbols. Deviations in the FSFs are plotted in the right column (b, d). The surface of the water phantom was positioned at isocenter without RaShi and at ISD50cm with RaShi in (a–b) and (c–d), respectively. [Color figure can be viewed at http://wileyonlinelibrary.com]

In contrast to the above described FSFs calculated from the *core* only, the fully MC simulated and measured FSFs, which include *halo* contributions, had an enlarged range and notably exceeded unity as can be seen in Figs. 7 and 8. FSFs considering the *core* only and the FSFs including the *halo* match best for the lowest energy (62.4 MeV, blue curves in Figs. 6 and 7), where the FSF range increased only by few percent. The deviations in the FSFs caused by the underestimated *core* width (dashed blue line in Fig. 6) using the Gaussian approximation agreed in magnitude and distribution to the FSFs including *halo* [blue curve in Fig. 7(b)]. Consequently, the major fraction of the deviations in the fully MC simulated to the measured FSFs of the lowest energy without RaShi can be attributed to the underestimated dose *core* width, $\sigma_{C}$. For the highest energy, the field size effects were higher including the *halo* and so were the deviations. The maximum FSF deviations were +3.6 and −2.3% at field sizes smaller than 4.4 cm at depths with the biggest $\sigma_{C}$ and at least partially caused by $\sigma_{C}$ uncertainties originating in MCS simulation and the beam model. For all energies without RaShi and *F* > 4.4 cm MC simulated FSFs were within the measurement uncertainty until around midrange. At 80 and 97% of the beam range (252.7 MeV), MC deviated significantly with respect to the measurement accuracy. Despite the reduced maximum deviation with RaShi, the two to three times higher RMSD listed in Table 3 compared to similar ranges in water without RaShi quantifies the worse agreement with RaShi. All RMSD at constant depths were lower than 1%, but two to three times higher in the last half of the beam range, compared to the first half. FFs and FSFs were overestimated for large frames/fields (*F* > 10 cm), whereas they were mostly underestimated for smaller fields/frames.

**Table 3:** Root‐mean‐square deviation (RMSD) of the FSFs at depth *z* relative to the beam range of energy *E* using *QBBC* (*δ*) or *QGSP*_*BIC* ($\delta_{\mathrm{BIC}}$), respectively. Only field sizes 4.4 cm ≤ *f* ≤ 20 cm were considered

	Open beam					RaShi			
*E* (MeV)	62.4	148.2		252.7		97.4		124.7	
*z* (%)	*δ*	*δ*	$\delta_{\mathrm{BIC}}$	*δ*	$\delta_{\mathrm{BIC}}$	*δ*	$\delta_{\mathrm{BIC}}$	*δ*	$\delta_{\mathrm{BIC}}$
$z_{0}$	0.3	0.1	0.1	0.1	0.1	0.8	0.4	0.5	0.2
50%		0.1	0.1	0.2	0.1			0.6	0.3
80%		0.2	0.1	0.6	0.3			0.6	0.3
97%		0.3	0.1	0.9	0.3			0.6	0.3

The FSFs at an approximately constant relative depth (80% and 89% of the beam range) for all measured five energies with and without RaShi are shown in Fig. 7. The 97.4 MeV beam after traversing the RaShi has approximately the same range in water as the 62.4 MeV beam without RaShi, but exhibits a FSF at 20 cm field size equal to 102.8%. The additional scattering of protons in the RaShi caused the approximately 2.4% higher FSF compared to the 62.4 MeV beam. The lowest range of FSFs was found for the medium energy beam (148.2 MeV) without RaShi. The highest energy without RaShi and the lowest energy with RaShi resulted in the highest FSFs, which is in consistency with the *halo* in the lateral dose profiles. While the high FSFs at large fields originate in scattering within the phantom without RaShi, it was scattering in the RaShi for the energies passing through it. The medium energy beam (148.2 MeV) without RaShi exhibited the lowest maximum and range of FSFs (see Fig. S2).

Figure 8 shows FFs and FSFs at four depths for two energies. At constant depths, measured FFs decreased steeply with increasing frame size for small frames (<8 cm), and leveled off at medium field sizes before they fell‐off again after 15.6 cm in the open beam configuration. The slope of the fall‐off of FFs for small frames was inversely proportional to the *core* width $\sigma_{C}$ (due to out‐scattering). The almost constant FFs at medium frame sizes were highest around midrange. The fall‐off at frames greater than 15.6 cm for all energies could origin in the steep fall‐off of elastic differential cross sections around $7^{\circ }$ 1, 34 as the lateral displacement at the isocenter of a proton scattered by $7^{\circ }$ in the vacuum exit window coincides with this value (≈16 cm). FFs were increasing from frame 10.0 to 15.6 cm due to the 4.6 times higher number of irradiated PBs (+2680 PBs) in the 15.6 cm frame at 50% and 80% of the range in the 252.7 MeV beam. Obviously, FFs become very small for large fields, but for all measured frames, FFs were greater than zero. Consequently, all FSFs monotonically increased with field size and exceeded 100% for field sizes larger than 10 cm. This effect may be negligible (up to 0.3%) for the 62.4 and 148.2 MeV beam without RaShi, but not for the 252.7 MeV beam or beams traversing the RaShi where FSFs exceeded unity at midrange by up to 5%.

The measurement uncertainty on FSFs [Δ*FSF* according to Eq. (5)] is represented as shaded area in Fig. 8 and was less than 0.5% although the standard deviation of the measured charge of the largest frame was up to 18% in three repetitive measurements. The Δ*FSF* increased noticeably in the smallest fields and remained basically constant for larger fields. FFs can approximately be thought of as a weighting factor for the error contributing to the FSFs.

Using the *QGSP*_*BIC* instead of *QBBC* resulted in a better agreement with measurements of the FSFs and FFs after midrange of the 148.2 and 252.7 MeV beams without RaShi, where the maximum underestimation decreased to −0.7% and the RMSDs reduced by a factor two to three. The underestimation of FSFs for small to intermediate field sizes (2 cm < *F* < 8 cm) was lower in *QGSP*_*BIC* and is consistent with the reduced overestimation of the lateral dose profile in the approximately 3 to 8 cm radius in Fig. 3 toward the end of range. With RaShi the accuracy improved at all depths and energies considerably by approximately a factor two in terms of RMSDs (see Table 3).

## 4 Discussion

In this study, the simulated values of $\sigma_{C}$ for the dose core agreed well with measurements for all energies. The modest overestimation of the beam broadening with depth compensated for the underestimated $\sigma_{C}$ in the beam model at the water phantom surface. Consequently, $\sigma_{C}$ agreed best at the Bragg peak, although the transformation to $\sigma_{\mathrm{sc}}$ revealed the overestimated $\sigma_{C}$ broadening toward the end of the range. This may be due to an overestimation of the MCS or the initial beam divergence in the beam model [see Eq. (2)]. As there was no systematic trend in $\sigma_{C}$ deviations over 78 cm range in air after the Nozzle exit,^19^ the beam divergence seems to be reasonably well modeled. Therefore, we associate the overestimated beam broadening with an overestimation of the MCS. A separate study^15^ reported on average over all materials an underestimation of scattering angles by 1.1%, where scattering angles increased strongly when the target thickness to range ratio reached 70%. This underestimation at small ranges and less underestimation toward the end of the range is consistent with the simulations using the non‐divergent beam in our study. Similar conclusions were found on the Geant4 MCS in an independent study.^35^

The lateral dose profiles agreed qualitatively well in the *halo* region in the open beam configuration but was substantially overestimated when traversing the RaShi. Due to the shallow gradient of the lateral dose profile in the *halo* after traversing the RaShi, FWxM parameters were considerably overestimated although *OFs* could be rather well reproduced. Therefore, large differences in FWxM do not necessarily result in large errors on *OFs*, particularly in regions with a low dose gradient.

The general good agreement of the dose *halo* profile without RaShi for all three energies provides evidence that there is no systematic problem at intermediate radii (3 and 6 cm) in Geant4, but support the misalignment hypothesis in a recent validation study using *QGSP*_*BIC*_*HP*.^17^

The deviations in the lateral dose profile and *OFs* were lower when using the *QGSP*_*BIC* toward the end of the range in the open beam configuration and high energies or when the beam traversed the RaShi. Taking into account that the two hadron physics lists are vastly similar in the clinical relevant energy range, these deviations are not self‐explanatory. Non‐elastic nuclear interactions are treated by the binary cascade model up to 1.5 and 9.9 GeV in *QBBC* and *QGSP*_*BIC*, respectively. When we replaced the default proton elastic CSs in *QBBC* (BGG) with the CSs used in *QGSP*_*BIC* (CHIPS), the accuracy improved to the level observed with the latter.

**Figure 9:**
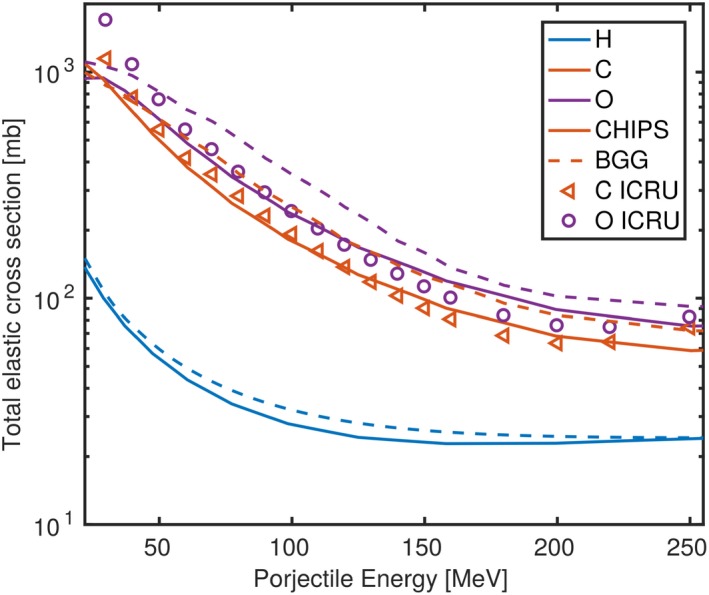
Elastic scattering CSs used in Geant4 compared to experimental values adopted from ICRU report 63.^37^ [Color figure can be viewed at http://wileyonlinelibrary.com]

The elastic scattering CSs of those two models are compared to experimental values in Fig. 9. The CSs obtained from BGG are approximately 10–50% higher compared to CHIPS for carbon and oxygen targets in the energy range from 60 to 250 MeV. Elastic scattering CS of proton projectiles on hydrogen targets deviated less (up to +10%). In this energy range CS from CHIPS agreed with experimental values within approximately ±20%, where the CSs were overestimated for energies exceeding 130 MeV and underestimated for lower energies. In the same regimen, CSs derived from BGG were 20 to 50% higher than the tabulated CSs. Therefore, we conclude that the major fraction of the observed deviations in the dose *halo* after traversing the RaShi or at the distal planes in water without traversing the RaShi originated in the handling of the elastic scattering.

Measured laterally integrated depth dose distributions (IDDs) of a central PB are required by some vendors to create a beam model for a specific beam line in a treatment planning system. Large area detectors such as the Bragg Peak Chamber (PTW, Freiburg) are used for such measurements to cover a large diameter. Yet, a considerable fraction of the dose remains undetected and needs to be corrected for by measurements^2^, ^14^ or MC simulations.^12^, ^13^ The radius of the Bragg Peak chamber (r = 40.8 mm) corresponds to a square field size equal to 7.2 cm (assuming the same area of the square and the circle of the active area). Simulated FSFs of this or bigger frame sizes were within the measurement uncertainty (using *QGSP*_*BIC*) for all tested energies. Consequently, we conclude that Geant4 can be used to correct for the missing dose of IDD distributions in the clinically relevant energy spectrum assuming a sufficiently accurate beam model.

The range in water of the lowest energy beam (62.4 MeV) is 3 cm in water. The PB before entering the water is of Gaussian shape; within 3$\sigma_{C}$ (≈3 cm) about 99.7% of all doses and approximately also particles should be found. Therefore, protons scattered within the water phantom cannot reach a lateral displacement of approximately 6 cm. Only protons scattered within the nozzle and neutral particles can reach lateral distances of that order. The 97.4 MeV beam after traversing the RaShi has approximately the same range in water as the 62.4 MeV beam without RaShi, but the FSF at 20 cm field size was approximately 2.4% higher. This origins in the additional scattering of protons in the RaShi and the subsequent 22 cm drift before entering the phantom. In the terminology used in Gottschalk et al. 2015:^1^ the RaShi causes additional *spray*. Nuclear scattering cross sections have their maximum approximately at 20 MeV and scattering angles increase with decreasing energy.^1^, ^36^ Therefore, *OFs* were higher for the lower energy with RaShi. Without RaShi this effect cannot be resolved due to the low *spray* contributions from the nozzle and the competing *halo*, the accumulation of particles scattered within the phantom. The maximum FSFs of the low‐ and medium‐energy beam without RaShi were similar. The *halo* dominated in the highest energy beam exhibiting the highest FSFs.

**Figure 10:**
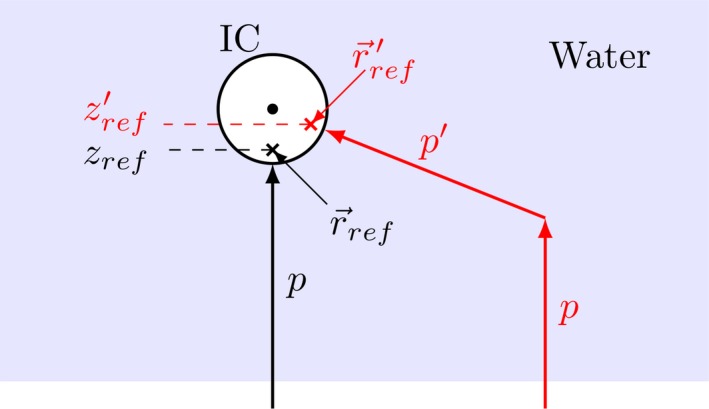
Schematic illustration of the shift of the effective point of measurement (${\vec{r}}_{\mathrm{ref}}^{\;\prime }$) of a cylindrical IC for a large‐angle scattered proton ($p^{\prime }$) with respect to the primary proton (*p*) beam direction. [Color figure can be viewed at http://wileyonlinelibrary.com]

In this study, *OFs* were measured with an air filled cylindrical IC (SF), where the geometrical center is shifted by Δ*z* = +2.1 mm in beam direction relative to the effective point of measurement ${\vec{r}}_{\mathrm{ref}}$ 23, 24 illustrated in Fig. 10. In contrary to measurements, dose was scored in a water filled cylinder centered at $z_{\mathrm{ref}}$ in the MC simulations. We assume that this results in two systematic errors. Firstly, for in‐field measurements (the 2 cm × 2 cm field) the particles impinge the water cylinder approximately parallel to the beam direction and slow down more than in an air filled IC. The diameter of the volume is 5 mm, which is important for the measurements in a high dose gradient in beam direction, for example, 13% mm${}^{-1}$ in the 62.4 MeV beam at $z_{\mathrm{ref}}\;=\;26.7$ mm or the distal planes for 148.2 MeV (10% mm${}^{-1}$) and 252.7 MeV (4% mm${}^{-1}$). Secondly, for out‐of‐field measurements most protons ($p^{\prime }$) impinge the IC at a large angle with respect to the primary beam direction. This is illustrated for a single large angle scattered proton in water in red in Fig. 10. The shift of the effective point of measurement is along proton propagation direction, hence when the particles impinge the IC at a large angle (with respect to the primary beam path), the effective depth of measurement should shift toward the geometrical depth of the detector, that is, away from the surface, for increasing field sizes. From geometry, this effect should increase with increasing lateral and decreasing axial distance of the scattering point to the IC (so with field size and inversely with distance from the entrance window). The shift of $z_{\mathrm{ref}}^{\prime }$ impacts OFs if there is a dose gradient along depth. FW0.1%M and FW0.05%M reveal an increasing low dose width of the 148.2 and 252.7 MeV beam relative to the central axis dose. In contrast to the high‐energy beams, the low energy beam without RaShi and the beams with RaShi do not exhibit a steep increase. Using an almost water equivalent dosimeter could possibly experimentally quantify the systematic inequality of FSFs measured with an IC and the FSFs at $z_{\mathrm{ref}}$ in water used in calculations, both MC as well as PB algorithms.^2^, ^37^

Validating MC simulations on lateral dose profile measurements is more intuitive than comparing *OFs*. However, in our experimental setup *OF* measurements suffered less from alignment accuracy and detection limits, which is discussed more thoroughly in the supplementary materials.

Measured and simulated FSFs in this study agreed well with three other studies^4^, ^5^, ^18^ for the highest energy, but differences up to 1% were observed for the largest field sizes compared to two other studies^3^, ^11^ (see Fig. S3).

The results of this work are valid for Geant4 based applications using version 10.3.p01 and compatibility to future releases should be validated as there are steadily developments.^9^, ^38^, ^39^

## 5 Conclusions

Simulated dose *core* profiles agreed within −7%/+2% with measurements. The relative broadening of the beam in water deviated negligibly from measurements until midrange. Toward the end of the range, the relative *core* broadening was overestimated for all energies. Consequently, one may purposely underestimate the beam width in the MC beam model at the entrance to increase the accuracy of the beam *core* width at the Bragg peak. This may be done without compromising the accuracy with respect to FSFs at high beam energies if the initial beam width is sufficiently small.

Simulated and measured dose *halo* profiles at constant depths agreed generally well in the open beam configuration, where only after midrange a moderate overestimation of the dose *halo* at intermediate radii (approximately 3$\sigma_{C}$–7$\sigma_{C}$) was observed. However, considerable overestimation of the dose *halo* was found when the beam traversed the RaShi, which could be tracked down to origin in the applied elastic scattering CSs.

The dose *halo* was considerably higher and calculation accuracy lower for beams traversing the RaShi compared to beams with similar ranges in water without traversing the RaShi.

The FF and FSF accuracy is influenced by both, the dose *core* and *halo*. FSFs up to a field size equal to approximately 4 cm can be largely influenced by small *core* modeling uncertainties. Therefore, the FSFs were evaluated by means of RMSDs for fields from 4.4 to 20.0 cm side length. MC simulated FSFs agreed well with measurements until midrange in the open beam configuration demonstrating that nonelastic nuclear scattering was well modeled. The accuracy decreased toward the end of the range in the open beam configuration or when the beam traversed the RaShi for both tested hadron physics lists. Consequently, applying the RaShi increased field size effects as well as reduced dose calculation accuracy.

In this study, FSFs deviations were approximately a factor of two higher using *QBBC*, where the nuclear elastic scattering CSs of protons caused a major fraction of the deviations. The BGG CSs used in *QBBC* were substantially higher than CSs in literature, whereas the CHIPS CSs (by default applied in *QGSP*_*BIC*) showed higher accuracy. Therefore, *QGSP*_*BIC* can be recommended for proton beam therapy simulations in Geant4 version 10.3.

The high accuracy of the simulated *OFs* suggest that the dose not detected due to the limited geometrical cross‐section of the commercially available large area detectors can be corrected by Geant4 simulations in the energy range from 62 to 252 MeV if the beam model is sufficiently accurate.

Field size effects were almost negligible for beams up to medium energy and were highest for the highest energy beam without RaShi, but vice versa when traversing the RaShi. Therefore, a finer measurement grid may be useful at high energies for beam lines similar to the one in this study, but reversely for beam lines exhibiting more *spray* from the nozzle or when a RaShi needs to be applied.

*OFs* are useful to quantify the accuracy of dose calculation in the low dose region, whereas dose and FWxM profiles may be better suited for qualitative evaluation. Measuring FFs instead of FSFs allows to exploit the different sensitivity ranges of the electrometer, which seems to be beneficial when the currents are close to the detection limit.

In this study, GATE/Geant4 simulations were validated on three types of dose measurements in the entire clinically relevant energy range. The observed deviations were consistent within the measurements and acceptably low in one of the two tested physics configurations.

## Acknowledgment

The authors thank the entire medical physics team at the MedAustron Ion Therapy Centre for their support and in particular Ralf Dreindl for providing additional data and helpful discussions. The financial support by the (Austrian) Federal Ministry for Digital, Business and Enterprise and the National Foundation for Research, Technology and Development is gratefully acknowledged.

## Conflict of Interest

The authors have no conflict to disclose.

## Supporting information


**Data S1:** Supplementary Materials.
