# Supplementary material for: Evaluation of electromagnetic and nuclear scattering models in GATE/Geant4 for proton therapy
Source: Med Phys. 2019 Apr 15;46(5):2444–56. doi: 10.1002/mp.13472 (PMC6850424; doi:10.1002/mp.13472)
Supplement: Supplementary file 1 — Data S1: Supplementary Materials. [file MP-46-2444-s001.pdf]

Supplementary materials to: Evaluation of electromagnetic and nuclear scattering models in GATE/Geant4 for proton therapy

A.F. Resch*

Division Medical Radiation Physics, Department of Radiotherapy, Christian Doppler Laboratory for Medical Radiation Research for Radiation Oncology, Medical University of Vienna/AKH Wien, Währinger Gürtel 18-20, 1090 Vienna, Austria

A. Elia

MedAustron Ion Therapy Centre/EBG MedAustron, Marie-Curie-Straße 5, 2700 Wiener Neustadt, Austria

H. Fuchs

Division Medical Radiation Physics, Department of Radiotherapy, Christian Doppler Laboratory for Medical Radiation Research for Radiation Oncology, Medical University of Vienna/AKH Wien, Währinger Gürtel 18-20, 1090 Vienna, Austria

A. Carlino

MedAustron Ion Therapy Centre/EBG MedAustron, Marie-Curie-Straße 5, 2700 Wiener Neustadt, Austria

H. Palmans

MedAustron Ion Therapy Centre/EBG MedAustron, Marie-Curie-Straße 5, 2700 Wiener Neustadt, Austria and Medical Radiation Science, National Physical Laboratory, Hampton Road, TW11 0LW Teddington, United Kingdom

M. Stock

MedAustron Ion Therapy Centre/EBG MedAustron, Marie-Curie-Straße 5, 2700 Wiener Neustadt, Austria

D. Georg

Division Medical Radiation Physics, Department of Radiotherapy, Christian Doppler Laboratory for Medical Radiation Research for Radiation Oncology, Medical University of Vienna/AKH Wien, Währinger Gürtel 18-20, 1090 Vienna, Austria

L. Grevillot

MedAustron Ion Therapy Centre/EBG MedAustron, Marie-Curie-Straße 5, 2700 Wiener Neustadt, Austria

**andreas.resch@meduniwien.ac.at*

S1. OVERVIEW OF THE MEASUREMENTS

TABLE S1. Overview of the experimental data. The nominal beam energy E, the number of delivered particles per spot (N_p), the isocenter to detector surface distance (ISD), whether or not the beam was passing a range shifter (RaShi), the detector used and the reference depth of measurement z_{ref} are listed for each measurement.

Type	Beam config.	E [MeV]	N_p / spot	ISD [cm]	RaShi	Detector	z_{ref} [mm]	Comment
Core	single spot	62.4	$5 \cdot 10^9$	0	no	MD	14	measured in vertical and horizontal direction, perpendicular to beam direction
		97.4	$5 \cdot 10^9$	0	no	MD	14, 56	
		148.2	$5 \cdot 10^9$	0	no	MD	20, 75, 120, 146	
		198.0	$5 \cdot 10^9$	0	no	MD	20, 125, 200, 244	
		252.7	$5 \cdot 10^9$	0	no	MD/PP	20, 190, 304, 371	
Halo	single spot	62.4	10^{10}	0	no	PP	27	measured in horizontal direction, perpendicular to beam direction
		148.2	10^{10}	0	no	PP	27, 73, 118, 146	
		252.7	10^{10}	0	no	PP	27, 188, 302, 369	
		97.4	10^{10}	50	yes	PP	27	
		124.7	10^{10}	50	yes	PP	27, 38, 60, 73	
OF	hollow frames	62.4	10^7	0	no	SF	27	9 hollow frames at each depth measured
		148.2	10^7	0	no	SF	27, 73, 118, 146	
		252.7	10^7	0	no	SF	27, 188, 302, 369	
		97.4	10^7	50	yes	SF	27	
		124.7	10^7	50	yes	SF	27, 38, 60, 73	

S2. ADDITIONAL RESULTS

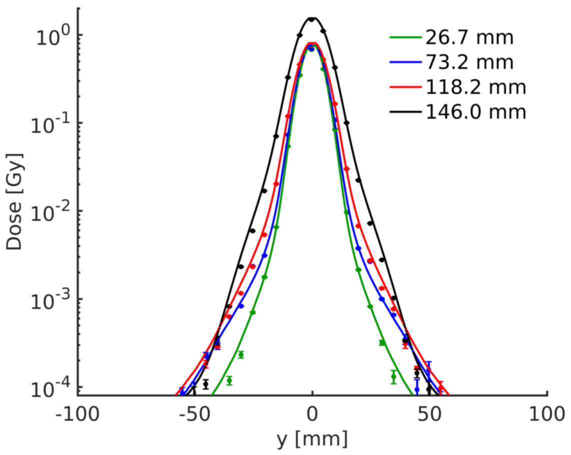

FIG. S1. Simulated (line) and measured (markers) lateral dose profile of the 148.2 MeV beam at four depths in water.

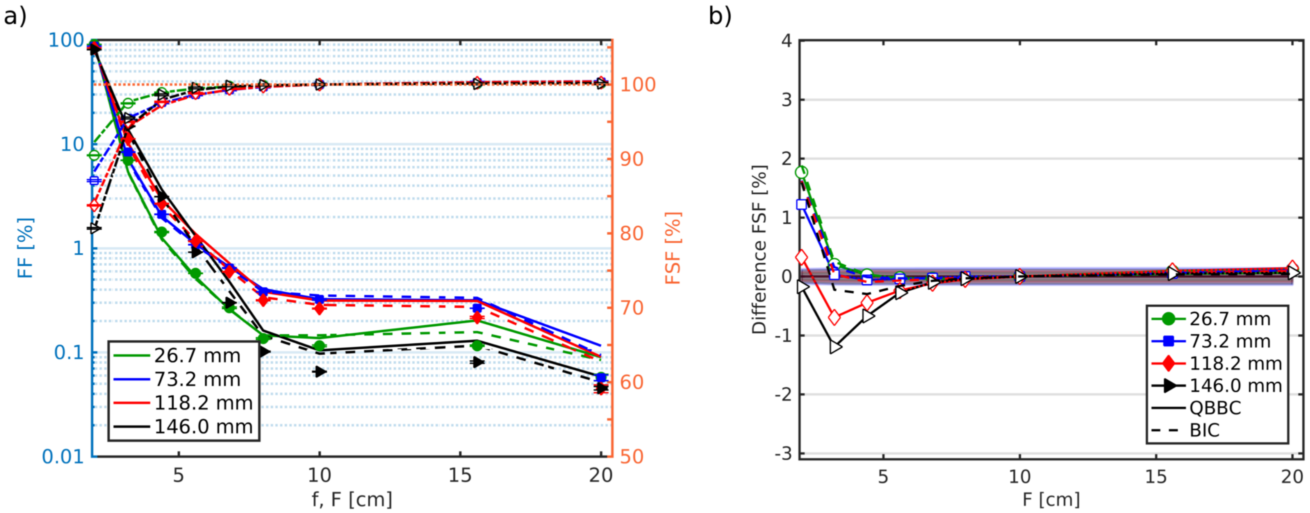

FIG. S2. FF and FSF as a function of field sizes for the 148.2 MeV beam are plotted in a). MC simulations are represented with lines and measurements with symbols. Deviations of the FSFs are plotted in b).

30 ## S3. COMPARISON TO LITERATURE

The *halo* of high energies around midrange is almost entirely produced within the phantom and Nozzle contributions are approximately negligible. Therefore, in FIG. S3 the FSFs of the highest available energy in literature (≈ 225 MeV) are compared to FSFs reported in this study. Since the highest energy measured in this study was approximately 25 MeV

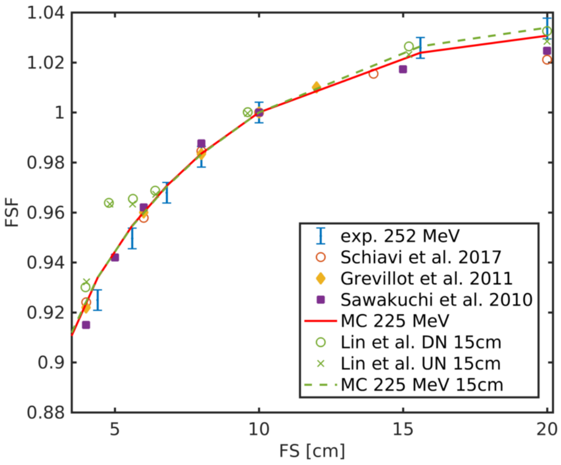

FIG. S3. Comparison of FSFs with literature for a high energy proton beam. The nominal energies were (225 ± 3) MeV at depths 15 cm (47% range) (Lin et al. UN, DN [1, 2]), 20 cm (61% range) (Grevillot et al. [5], Schiavi et al. [4]) and 23 cm (73% range) (Sawakuchi et al. [3]). FSFs were simulated using a nominal initial energy equal to 225 MeV at 15 cm (green) and 20 cm (red). Experimental FSFs of the 252.7 MeV beam at 50% range of this study are reported with errorbars.

higher than highest energies in literature, FSFs were derived from simulations using the same energy and $QGSP\_BIC$. The experimental results of the 252.7 MeV of this study are displayed to give an estimate on the confidence interval of those values. Comparing those simulated FSFs using the 27.7 MeV lower energetic protons, shows that there is no strong dependence on energy and, consequently, that the slightly different energies reported in literature are negligible. The agreement of all reported FSFs up to approximately 12 cm field size is excellent. The deviation of approximately 1% at the smallest field size may be addressed to different σ_C sizes. A trend of systematically higher FSFs determined at our and another facility [1, 2] (≈ 1 %) compared to two other facilities [3, 4] appears for the biggest two field sizes. Further investigations could clarify whether this deviation origins in *spray* from the individual nozzle configuration or in the applied detector. In our study a thimble IC (Semi-Flex) was used whereas parallel plate detectors were used in the two other studies (an Advanced- or a Markus Chamber, PTW, were employed in [3] and [4], respectively). Consequently, the differences could origin in the shift of effective point of measurement (see Fig 10 in the main article) or the different sensitivities of the detectors.

S4. EXPERIMENTAL AND METHODOLOGICAL LIMITATIONS

A. Set-up uncertainties

The alignment of the measurement setup with the in-room lasers resulted in a misalignment up to 0.7 mm in the horizontal measurements with the 24 PP ICs according to the Gaussian fit. The potential lateral displacement error in the order of 3 mm [6] is avoided by the use of the static linear array holder with constant spacing. The misalignment in vertical direction could not be determined, but is likely to be of similar magnitude. A vertical misalignment introduces an error to the FWxM determination, as the core is of Gaussian shape, but the low dose region decreases less steeply (more exponential like [7]). The influence of the misalignment can be reduced by applying spots along a line instead of a single spot [8]. The *core* and *OF* measurements are less sensitive to misalignment due to the Gaussian form and the symmetry of the field, respectively.

B. Leakage currents

The upper limit of the leakage currents are specified by the manufacturer (PTW) to be 50 fA and 1 fA for the Unidos in medium and low range, respectively. The leakage current from the SemiFlex IC does not exceed 4 fA according to specifications. Hence, a measurement time of 20 s or 3 min in medium and low range, respectively, results in a potential charge leakage equal to 1 pC. Measured FF charges ranged from 0.4 to 3 pC for frame sizes greater than 10 cm and energies 62.4 and 148.2 MeV, which were measured in low range setting of the Unidos webline. The measurement time of these large fields was of the order of a few minutes (2-3 min), which would correspond to a potential charge leakage up to 1 pC (30 to 300% of the measured charge). The measurement time of the inner field was about half a minute and carried out in medium range, which again is in the order of 1 pC potential leakage. However, we assume that leakage currents are stable over time and that they are partially accounted for by the zeroing of the electrometer. Furthermore, we assume that they are of random nature and do not introduce a systematic bias. This assumption is supported by one measurement of a 20 cm frame, which was measured in medium range and resulted in a 18% standard deviation in 3 measurements, which was at least double the deviation than comparable 20 cm frames measuring around 0.6 pC in low range. Standard deviations

of repeated measurements for the smallest field dominated the uncertainties of the FSFs (eq 5) and were in the order of 0.3%. Measuring the entire field size at once rather than the decomposed fields would require the medium range and, hence, supposedly result in higher uncertainties.

The leakage current of the Multidos (PP measurements) in low dose range is specified not to exceed 50 fA, which is of comparable magnitude as the 33 fA (0.5 pC/ 15 s) measured in [7]. In the PP measurements, 0.5 pC correspond to about 6×10^{-4} Gy, which may explain the increasing measurement uncertainty with decreasing dose. To reduce noise in the derived FW0.1%M and FW0.05%M, the dose profiles were fitted with an exponential function in a neighborhood of numerically closest values (±50%) to the desired dose level. This ensured smooth and stable FWxM computation, whereas any interpolating methods resulted in random oscillations.

---

[1] Liyong Lin, Christopher G Ainsley, and James E McDonough. Experimental characterization of two-dimensional pencil beam scanning proton spot profiles. *Phys. Med. Biol.*, 58:6193–204, 2013b.

[2] Liyong Lin, Christopher G Ainsley, Timothy D Solberg, and James E McDonough. Experimental characterization of two-dimensional spot profiles for two proton pencil beam scanning nozzles. *Phys. Med. Biol.*, 59(2):439–504, 2014.

[3] G O Sawakuchi, U Titt, D Mirkovic, G Ciangaru, X R Zhu, N Sahoo, M T Gillin, and R Mohan. Monte Carlo investigation of the low-dose envelope from scanned proton pencil beams. *Phys. Med. Biol.*, 55(3):711–21, 2010b.

[4] A. Schiavi, M. Senzacqua, S. Pioli, A. Mairani, G. Magro, S. Molinelli, M. Ciocca, G. Battistoni, and V. Patera. Fred: A GPU-accelerated fast-Monte Carlo code for rapid treatment plan recalculation in ion beam therapy. *Phys. Med. Biol.*, 62(18):7482–504, 2017.

[5] L. Grevillot, D. Bertrand, F. Dessy, N. Freud, and D. Sarrut. A Monte Carlo pencil beam scanning model for proton treatment plan simulation using GATE/GEANT4. *Phys. Med. Biol.*, 56(16):5203–19, 2011.

[6] David C Hall, Anastasia Makarova, Harald Paganetti, and Bernard Gottschalk. Validation of nuclear models in Geant4 using the dose distribution of a 177 MeV proton pencil beam. *Phys.*

Med. Biol., 61(1):N1–N10, 2016.

[7] Bernard Gottschalk, Ethan W Cascio, Juliane Daartz, and Miles S Wagner. On the nuclear halo of a proton pencil beam stopping in water. *Phys. Med. Biol.*, 60(14):5627–54, 2015.

[8] T Tessonnier, T T Böhlen, F Ceruti, A Ferrari, P Sala, S Brons, T Haberer, J Debus, K Parodi, and A Mairani. Dosimetric verification in water of a Monte Carlo treatment planning tool for proton, helium, carbon and oxygen ion beams at the Heidelberg Ion Beam Therapy Center. *Phys. Med. Biol.*, 62(16):6579–94, jul 2017.
